# Implementation of the patient version of the evidence-based (S3) guideline for psychosocial interventions for patients with severe mental illness (IMPPETUS): study protocol for a cluster randomised controlled trial

**DOI:** 10.1186/s13063-020-4200-z

**Published:** 2020-03-18

**Authors:** Johanna Breilmann, Reinhold Kilian, Steffi G. Riedel-Heller, Uta Gühne, Alkomiet Hasan, Peter Falkai, Andreas Allgöwer, Rainer Muche, Thomas Becker, Klemens Ajayi, Peter Brieger, Karel Frasch, Stephan Heres, Markus Jäger, Andreas Küthmann, Albert Putzhammer, Max Schmauß, Bertram Schneeweiß, Michael Schwarz, Markus Kösters

**Affiliations:** 1 Department of Psychiatry ll, Ulm University, Guenzburg, Bezirkskrankenhaus, Ludwig-Heilmeyer-Str. 2, 89312 Guenzburg, Germany; 2 Institute of Social Medicine, Occupational Health and Public Health, Medical Faculty, University of Leipzig, Leipzig, Germany; 3 Department of Psychiatry and Psychotherapy, University Hospital Munich, Munich, Germany; 4 Department of Psychiatry, Psychotherapy and Psychosomatics of the University Augsburg, Bezirkskrankenhaus Augsburg, University of Augsburg, Augsburg, Germany; 5 Institute for Epidemiology and Medical Biometry, Ulm University, Ulm, Germany; 6 Kbo-Isar-Amper hospital, Haar, Taufkirchen, Munich, Germany; 7 District hospital Donauwörth, Donauwörth, Germany; 8 District hospital Kempten, Kempten, Germany; 9 District Hospital Memmingen, Memmingen, Germany; 10 District Hospital Kaufbeuren, Kaufbeuren, Germany

**Keywords:** Guideline, Implementation, Patient version, Cluster randomised controlled trial

## Abstract

**Background:**

The German guideline on psychosocial interventions for people with severe mental disorders recommends a broad spectrum of evidence-based treatments. Structured implementation of the associated patient version of the guideline is missing to date. The study aims to assess whether structured implementation of a patient guideline improves the empowerment of patients with severe mental disorders, as well as knowledge, attitudes and experiences regarding psychosocial interventions, service use, treatment satisfaction, treatment needs, quality of life and burden of care.

**Methods:**

The study is a multicentre, cluster-randomised, controlled study with two parallel groups. Inpatients and day hospital patients (all sexes; 18–65 years) with severe mental disorders will be included. Additionally, relatives of patients with mental disorders (all sexes; ≥ 18 years) will be included. In the experimental group, the patient guideline will be implemented using a multimodal strategy. Participants in the control group will receive treatment as usual but will be made aware of the patient guideline. The primary outcome is the change of empowerment, assessed by using the ‘empowerment in the process of psychiatric treatment of patients with affective and schizophrenia disorders’ (EPAS) scale. In addition, knowledge, attitudes and experiences regarding psychosocial interventions will be assessed as secondary outcomes, as well as service use, satisfaction with care, patient need and quality of life and participation and social inclusion. For relatives, the perceived burden of care also will be recorded. Results will be analysed using hierarchical linear models. For the health economic evaluation, the incremental cost-utility ratios will be computed using the differences in total costs of illness and the differences in quality-adjusted life years (QALY) between study groups.

**Discussion:**

The study will be the first to assess the effects of a structured implementation of the patient version of a psychiatric treatment guideline. The study has some limitations regarding the transferability of the results to other patients and other regions. Furthermore, problems with the recruitment of patients and relatives and with the implementation of intervention could occur during the study.

**Trial registration:**

The study is registered in the German Clinical Trials Register (DRKS) and the WHO International Clinical Trials Registry Platform (ICTRP) under registration number DRKS00017577 (Date of registration: 23 October 2019.

## Background

Psychosocial interventions aim to improve the ability of individuals to live in their social environment and participate in society [1]. They are a core component in the treatment of persons with severe mental illness. A wide range of very different psychosocial interventions exist, e.g., occupational therapy, art therapy and supported employment [1]. In 2012 the first version of the evidence- and consensus-based (S3) guideline ‘Psychosocial interventions in severe mental disorders’ of the German Association for Psychiatry, Psychotherapy and Psychosomatics (DGPPN) was published and provided a comprehensive assessment and appreciation of the available evidence on the efficacy of psychosocial interventions for persons with severe mental illness [1]. In addition, an associated patient guideline that reproduces the recommendations in plain language has been prepared to promote active participation of the patients in their treatment [2]. Both versions of the guideline were updated in 2019 [1, 2]. However, evidence suggests that patients in Germany are informed insufficiently about these interventions and that utilization rates are low [3]. Furthermore, at least in Germany, the uptake of psychosocial interventions seems to depend more on regional structures than on evidence [4].

While the pathway of evidence synthesis and guideline development is sophisticated, evidence on the effects of implementing treatment guidelines for mental illness in clinical practice is sparse and inconsistent [5]. In particular, the effects of patient guidelines have not been studied rigorously. Currently, no controlled study exists that has put to test whether an implementation of a psychiatric patient guideline has an influence on treatment utilization or outcomes. Some encouraging evidence of positive effects of patient information or patient guidelines can be derived from studies using, e.g., patient brochures in guideline implementation [6].

The present project aims to shed light on the effects of a structured patient guideline implementation within a multicentre, cluster-randomised, controlled, non-blinded, superiority study with a 1:1 allocation.

### Objectives

#### Primary objective

The primary objective is to assess, while using the ‘empowerment in the process of psychiatric treatment of patients with affective and schizophrenia disorders (EPAS)’ scale, whether a structured implementation of the patient guideline ‘Psychosocial interventions in severe mental disorders’ improves the empowerment of patients with severe mental disorders with regard to their treatment.

#### Other objectives

In addition, whether the structured implementation of the patient guideline ‘Psychosocial interventions in severe mental disorders’ is able to achieve the following is also assessed:

1. improve knowledge, attitudes and experiences of the patients and relatives about available treatments
2. improve service use by patients with severe mental disorders
3. improve treatment satisfaction of patients with severe mental disorders and their relatives
4. change treatment needs and coverage of needs of patients with severe mental disorders
5. improved quality of life of patients with severe mental disorders and their relatives
6. cause the gain of one quality-adjusted life year (QALY) in comparison to treatment as usual (TAU) at a maximum willingness to pay (MWTP) threshold of between 0 and 100.000 €
7. reduce the burden of care of relatives of patients with severe mental illness

## Methods/Design

This protocol describes a cluster-randomised controlled study, which is the second part of a larger project that also includes a cross-sectional study in the first part [7]. In the first part, an assessment of the current status of the implementation of the S3 guideline recommendations for psychosocial interventions was conducted. Furthermore, this cluster-randomised controlled study will be supplemented by a separate qualitative study.

The protocol was prepared in accordance with the SPIRIT statement (*see* Additional file 1) [8].

### Study design and setting

The study is a multicentre, cluster-randomised, controlled, non-blinded, superiority study with two parallel groups. Randomisation will be performed as a stratified block randomisation [9] with a 1:1 allocation (*see* ‘Randomisation’ section). Data will be collected in 10 clinics for psychiatry and psychotherapy, which provide in- and outpatient psychiatric care to the inhabitants of Upper Bavaria and Swabia, Germany. The centres provide care for metropolitan catchment areas (Augsburg and Munich), middle-urban regions (Kempten and Memmingen) and rural regions (Donauwörth, Günzburg, Kaufbeuren, and Taufkirchen).

### Eligibility criteria

Inpatients and day hospital patients will be included if they have a severe mental disorder according to the definition of the S3 guideline ‘Psychosocial interventions in severe mental disorders’ (disease duration of ≥ 2 years and substantial impact on activities of daily life) [1]. However, in contrast to the S3 guideline, the inclusion criteria for the present study will be limited to patients with schizophrenia, schizotypal and delusional disorders (ICD-10 F2x) or mood [affective] disorders (ICD-10 F3x) to obtain a more homogenous sample [10]. Considerable consequences on the activities of daily life will be defined as a ‘Global Assessment of Functioning’ (GAF) [11] score ≤ 60 and a ‘Health of the Nation Outcome Scales’ (HoNOS) [12] fulfilling one of two conditions: (a) a score of ≥ 2 on one of the items of the subscale for symptomatic problems (items 6, 7 and 8) and a score of ≥ 2 on each of the four items of the subscale for social problems (items 9, 10, 11 and 12) or (b) a score of ≥ 3 on at least one of these items (9, 10, 11 or 12). Patients of all sexes will be included if they are between the ages of 18 and 65 years. Patients need to have sufficient capacity to understand the research project and to decide about their participation. In case of doubts about the ability to obtain consent of the patient, the responsible physician will decide on the capacity to consent. Legal representatives of patients (if available) will be informed about the study participation in case of consent from the patient.

To include the perspective of relatives of patients with mental disorders, relatives of all sexes will be included if they are aged over 18 years and if they are capable of understanding the research project. Relatives will only be included if they are not in current inpatient or day hospital mental health care.

### Recruitment procedure

**Fig. 1:**
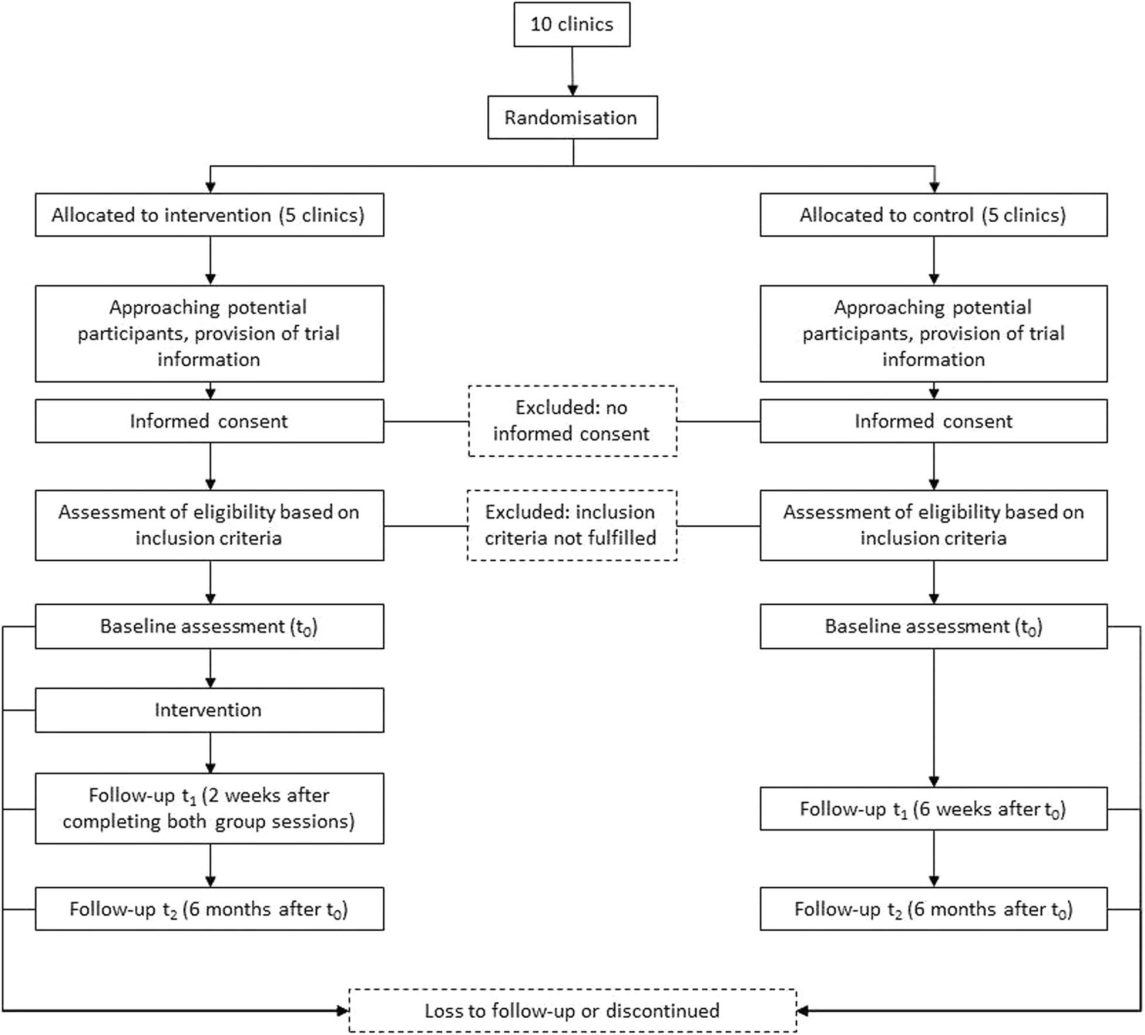
Flow of participants

The recruitment and data collection will be conducted from October 2019 to March 2021. Eligible patients will be approached and informed about the IMPPETUS study by the study staff to determine whether they would be willing to participate in the study, especially giving the consent to be willing to participate at a time point 6 months after intervention (t_2_). If the patients give their consent, a screening with the ‘Global Assessment of Functioning’ (GAF) and the ‘Health of the Nation Outcome Scales’ (HoNOS) will be carried out to identify patients with severe mental illness according to the definition above. The screening will be conducted as soon as possible after admission. For more details *see* Fig. 1.

Relatives will be addressed by the study staff in relatives groups and made aware by posters/flyers at the hospitals.

### Interventions

In centres randomised to the experimental group, the patient guideline ‘psychosocial interventions in severe mental disorders’ will be implemented using a multimodal strategy. The core component will be a two-part group session for patients of more than 60-min duration in which the contents of the patient guideline will be clearly presented (e.g., with case examples). The group session will be carried out jointly by the study staff (e.g., a psychologist) and a peer tutor (person with a lived experience of mental illness). The group sessions will also pay attention to the availability of psychosocial interventions in the regions but will not be limited to regional offers. In addition, questionnaires and decision aids on the basis of the patient guideline will be developed to assist patients in their discussions with practitioners and to promote their active demand for suitable psychosocial therapies. Training for the use of the questionnaires and decision aids will be provided in group sessions. Individual reminders will be developed. The two-part group sessions will be offered weekly in the inpatient and day hospital setting. Participation in both parts of the event will be supported by written information flyers. Repeated participation is possible, but study participants should attend both parts at least once. Furthermore, participants will receive the patient guideline as a printed book. Essential contents of the patient guideline will be made available on the Internet and will be optimized for mobile use (www.thera-part.de). The group sessions will include a brief introduction on how to use ‘TheraPart’. The plain language materials will be developed or tested with the participation of representatives of patients and relatives. A on-part information event for relatives will occur parallel to the two-part group events for the patients.

Patients in centres randomised to the control condition will receive treatment as usual but will be made aware of the patient guideline ‘Psychosocial interventions in severe mental disorders’ by receiving the same guideline flyers as participants in the intervention group, which summarises the key information of the guideline and refers to additional sources of information. Treatment as usual (TAU) was chosen as the control group to compare the intervention with standard treatment. Participating relatives of patients in clinics randomised to the control condition will also receive the flyer for the guideline.

Prior to the start of the study, all study staff will receive comprehensive training on the implementation of the assigned study conditions to ensure a consistent procedure across all sites. Regular retraining and supervision of the study staff will also take place. Regular exchange between all study staff and the study lead will ensure compliance with the study protocol.

### Outcomes and measurement

#### Patient-relevant outcomes and measurements

##### Primary outcome

The primary outcome is the improvement of empowerment (*see* objective 1) after 6 months, which will be measured as change according to the ‘empowerment in the process of psychiatric treatment of patients with affective and schizophrenia disorders’ (EPAS) scale [13]. The scale comprises a basic module with 33 items, which include the five dimensions ‘everyday coping‘, ‘social relationships‘, ‘treatment participation‘ and ‘hope and self-efficacy‘. Two add-on modules also cover empowerment in the care of underage children and at work. The questionnaire, whose psychometric properties have been successfully tested in a field study [13], provides a comprehensive record of empowerment in the psychiatric treatment process. Patients and their relatives will be interviewed with the EPAS.

##### Other outcomes

Changes in patient knowledge, attitudes and experiences regarding available treatments (*see* objective 2) will be assessed using a checklist developed for this study. The checklist includes all psychosocial interventions of the S3 guideline ‘psychosocial interventions’ (can be provided on request) and address the following:

1. Changes in service use (*see* objective 3) will be measured as changes on the German version of the ‘Client Sociodemographic and Service Receipt Inventory’ (CSSRI) [14]. The CSSRI is a semi-structured interview to assess social and demographic data, accommodation data, detailed information regarding treatment, professional visits and social and health service utilisation for estimating healthcare costs. Additionally, the CSSRI systematically records the use of psychiatric, medical, psychosocial and rehabilitative health services (direct costs) and productivity losses (indirect costs) and therefore completely covers the costs of the disease from an economic perspective. Therefore, the health economic evaluation (see objective 6) will be based on the data collected with the CSSRI. An appropriate pre-structuring of the requested health care services will ensure that the receipt of the psychosocial care services recommended in the S3 guideline ‘Psychosocial interventions’ will be assessed.
2. Satisfaction with treatment (*see* objective 4) of the patients will be assessed with the German satisfaction questionnaire ‘ZUF-8’ [15]. The ZUF-8 has eight items and is a one-dimensional measure of satisfaction to record the general or overall patient satisfaction with the treatment received. The item values are summed. High scale values indicate a large level of satisfaction and low scale values a low ‘satisfaction‘ (range from 8 to 32).
3. Changes in patient treatment needs and coverage of needs (*see* objective 5) will be assessed by using the German version of the ‘Camberwell Assessment of Need’ (CAN-EU) [16, 17]. The CAN-EU assesses 22 domains of potential individual care needs. Each domain is rated on a 3-point scale from the absence of need (= 0) to the presence of an unmet need (= 2). A total score is calculated by adding the domain ratings.
4. Quality of Life (QoL; *see* objective 6) of the patients will be measured with the German version of the World Health Organization’s Quality of Life Instrument-abbreviated version (WHOQOL-BREF) [18, 19]. The WHOQOL-BREF is a tool with 26 items: 24 items in four domains: physical (seven items), psychological (six items), social (three items) and environmental (eight items), and two additional items: the overall quality of life (item 1) and level of satisfaction with health (item 2). The items are rated on a five-point Likert-type scale. To calculate domain scores, the mean of all items within a domain is multiplied by four. Subsequently, these raw domain scores are transformed into a scale from 0 to 100 points (to enable comparisons to be made between domains composed of unequal numbers of items), with higher scores denoting better QOL.
5. The calculation of quality-adjusted life years (QALYs; *see* objective 7) will be based on the German Version of the EuroQol (EQ-5D-5 L) [20–22]. The EQ-5D-5 L generates health states at five dimensions as a basis for the generation of QALYs. The EQ-5D-5 L questionnaire includes five questions on current issues in the dimensions of ‘mobility‘, ‘self-care‘, ‘usual activities‘, ‘pain/discomfort‘ and ‘anxiety/depression‘. Five possible answers exist for each dimension: (1) no problems, (2) slight problems, (3) moderate problems, (4) severe problems and (5) unable to do. QALYs will be generated by means of the German value set [23].
6. In addition, the participation and social inclusion of the patients will be assessed using the German questionnaire ‘Fragebogen zur Erfassung sozialer Partizipation und sozialer Inklusion chronisch psychisch erkrankter Menschen’ (F-INK) [24]. The F-INK measures the participation and social inclusion of individuals with a chronic mental disorder. It is a modular questionnaire with nine modules that assess the central dimensions of social inclusion [24].
7. Sociodemographic data of the patients (e.g., sex, age and socioeconomic data) and patient medical history will be assessed within the CSSRI. The patient’s medical history (diagnosis, length of illness, length of current disease episode, number of previous episodes and current somatic and psychotherapeutic treatment) will be derived from the patient or the patient’s record data.

#### Relatives—relevant outcomes and measurements

Relevant outcomes and measurements for relatives include the following:

1. The mental disorder, the disorder severity and the restrictions in daily life of the participants’ mentally ill relatives will be assessed with a separate questionnaire.
2. Changes in the relatives’ knowledge, attitudes and experiences regarding available treatments (*see* objective 2) will be evaluated using the same developed checklist that was used for patients.
3. Satisfaction with treatment (*see* objective 4) from the perspective of the relatives will be assessed with the ZUF-8 (*see* above).
4. Changes in treatment needs and coverage of needs of the mentally ill relatives (see objective 5) will be assessed by using the CAN-EU (*see* above).
5. Quality of Life (QoL) of relatives of patients with severe mental illness (see objective 6) will be measured with the WHOQOL-BREF (*see* above).
6. Relatives’ burden of care (*see* objective 8) will be assessed using the ‘Burden Assessment scale’ (BAS) [25]. The BAS is a 19-item scale that focuses on specific subjective and objective consequences of relatives caring for persons with severe mental disorders within the past 6 months. Answers are given on a 4-point Likert scale ranging from (1) not at all to (4) very much, with higher values indicating stronger burden.
7. Sociodemographic data of the relatives will be assessed with a separate questionnaire.

### Data collection and management

Patients and relatives will be interviewed by the study staff at three time points: shortly after admission (baseline, t_0_), 2 weeks after visiting both parts of the group sessions (intervention group) or 6 weeks after t_0_ (control group) (t_1_), and 6 months after t_0_ (t_2,_ both groups) (*see* Table 1). Due to the large number of outcomes to be measured, the interview can, if necessary, be divided into several appointments in order to avoid excessive demands on the participants. The collected data will be recorded on paper and subsequently entered in a standardized data form at the recruiting centres. Data entry will be done by one study member and checked by another member (double check) to avoid mistakes. Data will be stored in a secured computerised database centrally at the study centre Günzburg. Participant files will be maintained in storage for a period of 10 years after completion of the study.

**Table 1:**
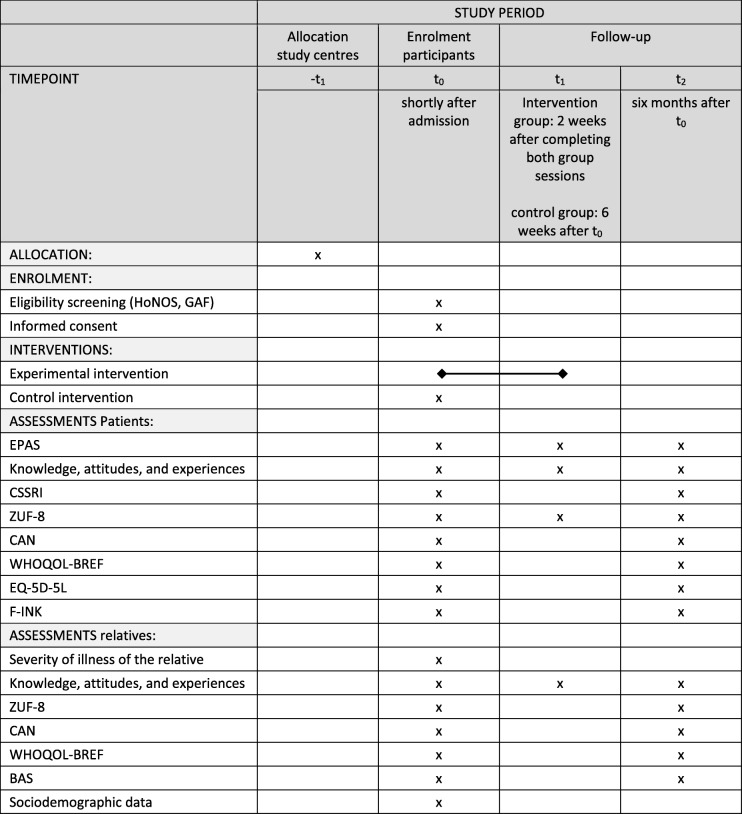
Template for the schedule of enrolment, interventions and assessments

To avoid follow-up losses, study staff will contact the participants regularly, for example by visiting the subjects during their inpatient stay. After discharge, they will be informed by newsletter and will be informed by phone or (e)-mail about upcoming data collection. Furthermore, the participants will be offered interview appointments after discharge that can be carried out at the participant’s home or by telephone.

Participants may withdraw from the study for any reason at any time. Participants who do not follow the intervention schedule will be interviewed as scheduled during the follow-up appointments. If possible, reasons for dropout are recorded.

### Data monitoring

A data monitoring committee was not established because no risks are to be expected for the participants in the intervention or the control group.

### Quality control

Prior to the start of the study, the study staff in all recruiting clinics will receive comprehensive assessment training. This rigorous training and regular meetings during the recruitment phase will ensure adherence to the protocol by all researchers involved in the study. During all study phases, the study lead will supervise the study.

### Sample size

Sample size calculation was based on a two-step procedure due to the hierarchical data structure. In the first step, the sample size for an individual randomized study is calculated. Based on studies using the ‘empowerment in the process of psychiatric treatment of patients with affective and schizophrenia disorders (EPAS)’ scale [26], a medium effect (0.4 SD units) of the implementation intervention on the primary endpoint is expected. This effect is detectable with an error probability of 5% (alpha) and a power of 80% by a t-test for independent samples with a sample size of 2 × 100 participants (200 in total).

The second step takes the cluster structure of the data into account. We assume that each centre recruits 35 patients (m) on average. The design effect (DE) is needed for the inflation of the group sample size: DE = 1 + (m - 1) * ICC with the fixed cluster size m and the intra-cluster correlation coefficient ICC. We assume a liberal intra-class correlation coefficient of 0.02 to control for the correlation of the outcome of participants of the same centre. Thereby, the DE is 1 + (35–1) * 0.02 = 1.68, which gives a sample size of 200 × 1.68 = 336 patients. As every centre recruits 35 patients on average, 10 centres are needed for the trial. Due to the severity of the disease in the patient population, case number planning assumes that 70% of patients can be reached 6 months after t_0_, so that an oversampling to (35/0.7) 50 patients per study centre will be recruited, resulting in 10 centres * 50 patients = 500 participants (2 × 250) as the overall sample size. This 70% estimate is based on experiences at the Günzburg site from three studies conducted with similar populations [27–29].

### Randomisation

The randomisation of the centres (cluster) to the intervention or control group will be done externally by the Institute for Epidemiology and Medical Biometry, Ulm University, Ulm, Germany, by a stratified block randomisation. The 10 recruiting centres will be randomised 1:1 to the intervention and control group using the size of centre (small, large) as strata. The random assignment will be performed by the randomisation program ROM [9].

### Statistical methods

First, the sociodemographic and clinical characteristics will be listed. Categorical variables will be described as absolute and relative frequencies and with graphical representation (e.g., bar chart). Continuous variables will be described by means and SDs, supplemented by minimum, median, percentiles and maximum. In addition, data will be analysed graphically (e.g., boxplot). Number of missing values will be reported for all variables of interest. Cases with missing values will be compared with those with complete data with regard to characteristics relevant for study outcomes.

The primary outcome is the improvement of empowerment 6 month after t_0_ (EPAS-t_2_) compared to baseline (EPAS-t_0_). Therefore, a hierarchical linear model (mixed model approach [30, 31]) is used to model EPAS-t_2_ as outcome and intervention, baseline EPAS-t_0_, centre and centre-size as main regressors with special attention to the intervention (confirmatory). The significance level will be set to 5% for intervention. The analysis will be based on a modified intention-to-treat (modITT)-population, using all patients with an outcome at month six (EPAS-t_2_) [32], since an imputation of the primary outcome can be misleading. Data management procedures will be implemented to reduce the drop-out rates; thus, a relatively low drop-out rate is expected. To examine a possible selection bias, the baseline data of ITT and modITT populations will be compared. In case of differences, these variables will be included in the regression models to adjust for confounding. For the independent variables, mixed model approaches can handle missing data well, and thus, no multiple imputation is needed in particular. However, according to this modITT concept, multiple imputation [33] of missing data in independent variables will be conducted by fully conditional specification (FCS) to perform a sensitivity analysis.

Other possible confounders with respect to the main outcome will be investigated by adding them into the model, eventually using backward selection procedures for variable selection and multiple imputations (*see* above). These analyses will be subjected to explorative interpretation only.

All other outcomes (*see* chapter ‘Outcomes and measurement’) will be analysed in a similar way. Subgroup analyses will be conducted separated by recruiting centres. Patient-related analyses (knowledge, attitudes, and experiences, service use, satisfaction with treatment, treatment needs and quality of life) will be conducted separately for patients with F2x and F3x diagnoses in additional subgroup analyses. Other categorisations based on quantitative variable are not planned.

Health economic evaluation (HEA) will be conducted from the societal and from the payer (statutory health insurance) perspective. Incremental cost-utility ratios (ICUR) will be computed using the differences in total (direct and indirect) costs of illness and the differences in quality-adjusted life years (QALY) between study groups over 12 months for the societal perspective. For the HEA from the payer perspective only the direct costs reimbursed by the statutory health insurance will be used as ICUR numerator. For both perspectives the stochastic uncertainty of the ICUR will be estimated by means of nonparametric bootstrapping with 2000 replications [34]. Probability of cost-effectiveness at MWTP for the gain of one QALY in the range between 0 and 100,000 € will be estimated for both perspectives by means of the cost-effectiveness acceptability curve [34]. The calculation of the QALY is based on the EQ-5D-5 L by means of the German value set [23].

All analyses will be conducted separately for patients and relatives.

### Ancillary and post-trial care

The subjects enjoy insurance protection while participating in the research project. The Ulm University Hospital and its employees involved in the study (study doctors, other staff) are covered by liability insurance if the test subjects are harmed through their fault. The subjects are also insured for accidental travel to and from the study centre.

### Amendments

In the event of protocol amendments, the date of each amendment will be accompanied by a description of the change and the rationale.

### Sponsor

Ulm University Medical Centre, Albert-Einstein-Allee 29, 89,081 Ulm, Germany is the trial sponsor. The sponsor is not involved in the design of the study, manuscript writing or collection of data, and the sponsor will not be involved in data analysis or interpretation and manuscript writing in the future.

### Dissemination

The results of this protocol study will be published in peer-reviewed journals as well as at national and international conferences. A publication and authorship agreement between all project partners was agreed, which regulates the procedure for publications in detail.

### Access to data

Principal Investigators will be given access to the cleaned data sets under conditions defined in the publication agreement. Analysis data sets do not contain identifying participant information.

## Discussion

This study is the first to assess the effects of a structured implementation of a patient version of a psychiatric treatment guideline. The results will shed light on the effects of a structured implementation of such patient guidelines and will provide insights how patients guidelines can be implemented.

Some limitations of the study should be noted. For example, recruitment will take place in clinics; thus, only patients with inpatient stays will be recorded. Furthermore, the assessment is limited to south-east Germany (Bavaria), so transferability of the results to other regions needs to be considered. In addition, the study will not cover the full range of patients with severe mental disorders, such as severe personality disorders (F60 - F61), severe anxiety disorders (F41) or severe obsessive-compulsive disorder (F42).

In addition, an imbalance may occur in the recruitment numbers between the two randomised groups, as the recruitment of subjects in one of the two groups could be more difficult than in the other (e.g., if the subjects do not want to participate without intervention). Therefore, the extension of the recruitment period may be necessary for one group to reach comparable sample sizes.

In the intervention group, difficulties may occur in maintaining the participation for both group sessions.

Furthermore, recruiting relatives may be difficult because relatives are often not easily accessible.

## Trial status

Protocol Version: 1.3, Date: 14. October 2019;

Date of first enrolment: 28. October 2019;

Estimated date of the end of recruitment: 31. March 2021.

## Supplementary information

**Additional file 1.** SPIRIT 2013 Checklist: Recommended items to address in a clinical trial protocol and related documents.

## Data Availability

The datasets used and analysed during the current study will be available from the corresponding author on reasonable request. Informed consent materials are only available in German. They are available on request from the corresponding author.

## Abbreviations

BAS: Burden Assessment scale
CAN-EU: Camberwell Assessment of Need
CSSRI: Client Sociodemographic and Service Receipt Inventory
DE: Design effect
DGPPN: German Association for Psychiatry, Psychotherapy and Psychosomatics
EPAS: Empowerment in the Process of Psychiatric Treatment of Patients with Affective and Schizophrenia Disorders Scale
EQ-5D-5 L: EuroQol
FCS: Fully conditional specification
F-INK: Questionnaire to Assess the Participation and Social Inclusion of the Patients
GAF: Global Assessment of Functioning
HEA: Health Economic Evaluation
HoNOS: Health of the Nation Outcome Scales
ICC: Intra-cluster correlation coefficient
ICUR: Incremental cost-utility ratios
IMPPETUS: Implementation of the Patient Version of the Evidence-based Guideline for Psychosocial Interventions for Patients with Severe Mental Illness
modITT: Modified intention-to-treat
MWTP: Maximum willingness to pay
QALY: Quality-adjusted life year
S3 guideline: Evidence and consensus-based guideline
SD: Standard deviation
t_0_: Assessment shortly after admission (baseline)
t_1_: Assessment 2 weeks after visiting both parts of the group sessions (intervention group) or 6 weeks after t_0_ (control group)
t_2_: Assessment 6 months after t_0_
TAU: Treatment as usual
WHOQOL-BREF: World Health Organization’s Quality of Life Instrument-abbreviated version
ZUF-8: German satisfaction questionnaire
